# Life expectancy of older people living in aged care facilities after a hip fracture

**DOI:** 10.1038/s41598-021-99685-z

**Published:** 2021-10-12

**Authors:** Enwu Liu, Maggie Killington, Ian D. Cameron, Raymond Li, Susan Kurrle, Maria Crotty

**Affiliations:** 1 Mary Mackillop Institute for Health Research, Australian Catholic University, Melbourne, VIC Australia; 2 College of Nursing and Health Sciences, Flinders University, Adelaide, SA Australia; 3 John Walsh Centre for Rehabilitation Research, Faculty of Medicine and Health, Kolling Institute, University of Sydney, St Leonards, NSW Australia; 4 Monash School of Medicine, Monash University, Melbourne, VIC Australia; 5 Curran Ageing Research Unit, Faculty of Medicine and Health, Hornsby Ku-Ring-Gai Hospital, University of Sydney and, Hornsby, NSW Australia; 6 College of Medicine and Public Health, Flinders University, Adelaide, SA Australia

**Keywords:** Diseases, Health care, Medical research, Risk factors

## Abstract

To the authors’ knowledge, no study has been conducted on life expectancy for aged care facility residents with hip fracture. We assessed life expectancy of 240 residents of aged care facilities in Australia who experienced recent hip fracture treated with surgery. 149 deaths occurred over a mean follow-up of 1.2 years. Being female and having better cognition were associated with longer life expectancy. Increased age was associated with shorter life expectancy. The cumulative mortality rate within three months after hip fracture was 25.0% while the cumulative mortality rate for the whole study period was 62.1%. Life expectancy was 8.2 years, 4.8 years and 2.8 years for 70, 80 and 90-years old female patients. Life expectancy was 3.8 years, 2.2 years and 1.3 years for 70, 80 and 90 years old male patients, respectively. In conclusion, age, gender and cognition level were associated with life expectancy of hip fracture patients living in aged care facilities and their life expectancy was much shorter than that of the general Australian population.

## Introduction

Hip fracture has a substantial impact on survival and health-related quality of life of older people^1,2^. It is estimated that 1.6 million hip fractures occur worldwide each year and by 2050 this number could reach between 4.5 million and 6.3 million^3,4^. Hip fracture poses a significant economic burden worldwide^5^. Across Australian and New Zealand, more than 25,000 people break their hip each year with an estimated cost of 1 billion dollar annually^6^. Life expectancy is a statistical measure of the number of years that a human expects to live based on one’s current age and other demographic factors. Life expectancy is a key indicator used to assess mortality trends, disease burden, overall health status of a population and monitor trends in health care over time used by the World Health Organization (WHO)^7^. In Australia people who are 65 years of age or older (50 years or older if identified as an Aboriginal or Torres Strait Islander person) are eligible for aged care services, the Australian government pays aged care service providers to deliver aged care through subsidies and supplements, capital grants for residential aged care and program funding^8^. In 2017–18, 7% Australian aged 65 and over were in residential aged care facilities and about one-quarter (27%) hip fractures occurred in aged care facilities^9^. While it is well known that hip fracture is associated with increased mortality in both sexes^10–12^, to the authors’ knowledge, no study has been conducted on life expectancy for aged care facility residents with hip fracture. For hip fracture patients living in aged care facilities knowing his/her life expectancy many help patients, family members and health care providers make plans and decisions for patients’ care over the remaining lifespan.

The Southern Adelaide Co-ordinated Regional Hip and Debility Rehabilitation Programme to Improve Quality of Life (SACRED) was a randomised clinical trial which examined whether providing rehabilitation in aged care facilities for people who were recovering from hip fracture surgery improved quality of life and mobility at 4 weeks and 12 months. The primary outcomes of the trial were mobility and quality of life. The main study was conducted between June 2012 and December 2014, for mortality, the last follow up was extended to December 2015. The detailed descriptions of the trial, intervention methods and main results were reported elsewhere^13,14^. The objective of this analysis is to assess the life expectancy of people 70 and older after hip fracture and identify factors influencing the survival time.

## Methods

### Study design and participants

We performed secondary analyses using the data from the Southern Adelaide Co-ordinated Regional Hip and Debility Rehabilitation Programme to Improve Quality of Life trial (SACRED). The trial was conducted between June 2012 and December 2014; for mortality data, the last follow up was 18 December 2015. Participants were medically stable residential aged care facility residents aged 70 years or older who had experienced a new, surgically treated hip fracture and were ambulant prior to their fracture either without assistance, with aids, or with the assistance of one other person. Participants unable to provide informed consent or obtain consent from a suitable proxy, had pathological and peri-prosthetic fractures, had a terminal illness and were receiving palliative care, had a hip fracture treated non-surgically or were unable to follow a one-step command due to cognitive impairment at recruitment were excluded from the study^13^.

### Outcomes

The outcome variable was survival time after hip fracture, calculated as date of death (all cause) or date of last follow up minus date of hip fracture. For patients surviving past 18 December 2015, the censored follow up time was calculated as 18 December 2015 minus the date of hip fracture. For patients who were lost to follow up or withdrew, survival time was calculated as date of last contact minus date of hip fracture.

### Covariates

All covariates were measured at baseline: age; BMI; cognition as measured by Mini-Mental State Examination (MMSE); delirium; any previous fractures; previous hip fractures; surgery types and randomised group. All measurements and data collection were performed by the researchers or trained nurses for the trial.

### Statistical analyses

Kaplan–Meier plots and log-rank tests were used to compare survival distributions across different groups. Weibull accelerated failure time (AFT) regression was chosen to investigate the associations between covariates and the survival time. Weibull AFT model was used to calculate life expectancy as it can produce robust results in ageing research^15^. The Weibull AFT model was specified as:

$${\mathrm{log}}\left ({t}_{i}\right)={\beta }_{0}+{{\beta }_{1}}{x}_{i1}+\dots +{\beta }_{p}{x}_{ip}+\sigma {\varepsilon }_{i}={{\varvec{x}}}_{{\varvec{i}}}^{\boldsymbol{^{\prime}}}{\varvec{\beta}}+\sigma {\varepsilon }_{i}$$

where subject i $(\mathrm{i}=\mathrm{1,2},\dots \mathrm{n})$ had $\mathrm{p}$ covariates ${x}_{i1,} {x}_{i2,}$ …${x}_{ip}$ and possibly censored survival time ${t}_{i}$, $\upsigma$ was the scale parameter, ${\varvec{\beta}}=\left ({\beta }_{0},\dots , {\beta }_{p}\right)$ were the regression coefficients of covariates, ${\varepsilon }_{1}\dots {\varepsilon }_{n}$ were independent and identically distributed according to the Gumbel distribution^16,17^. Life expectancy was calculated as the expected value of survival time $,\mathrm{ E}\left (\mathrm{T}\right)=\mathrm{exp} ({{\varvec{x}}}_{{\varvec{i}}}^{\boldsymbol{^{\prime}}}{\varvec{\beta}})\Gamma (\sigma +1)$ where the Gamma function has the form $\Gamma \left (\mathrm{z}\right)={\int }_{0}^{+\infty }{x}^{z-1}{e}^{-x}dx$^18^.

For sensitivity analysis, a Cox proportional hazards regression model was used to investigate the associations between covariates and mortality and results were compared with the results of the Weibull AFT model.

Analyses were performed using SAS 9.4 (SAS Institute, Cary, NC) and RStudio 1.2.5001 with R 3.6.3 (RStudio, Inc. Boston, MA).

### Ethics approval

The SACRED trial was registered on the Australian and New Zealand Clinical Trials Registry, registration number: ACTRN12612000112864. The Southern Adelaide Clinical Human Research Ethics Committee granted ethics approval for the secondary analysis of the trial and granted a waiver of informed consent (Application Number: 276.20). All research activities were carried out in accordance with the guidelines and regulations of the Australian National Health and Medical Research Council (NHMRC)**.**

## Results

A total of 240 participants of whom 178 (74.2%) were female and 62 (25.8%) were male were included in the study. The mean baseline age of participants was 88.6 (SD, 5.6) years, ranging from 70 to 101 years. The mean baseline mini-mental state examination (MMSE) score was 8.0 (SD, 7.8); 7.8% of participants had normal cognition or mild cognitive impairment, 33.8% suffered moderate cognitive impairment and 58.7% suffered severe cognitive impairment. 34.6% participants had delirium at baseline. The mini-nutritional assessment score was 5.3 (SD, 2.3). The mean BMI was 25.2 (SD, 4.9) kg/m^2^ with 5.4% underweight, 46.6% normal, 31.2% overweight and 16.7% obese. Ninety-four participants had experienced a previous fracture of any type and 32 had experienced a previous hip fracture. The most common surgery was intramedullary nailing (36.3%) and three participants had total hip replacement. Table 1 shows the baseline characteristics of participants.

**Table 1:** Baseline characteristics of the study population.

Baseline characteristics	Whole population (n = 240)
**Group**	
Intervention	119 (49.6)
Control	121 (50.4)
**Gender**	
Female	178 (74.2)
Male	62 (25.8)
**Age-years, mean (SD)**	88.6 (5.6)
70 to 79	16 (6.6)
80 to 89	118 (49.2)
90 to 101	106 (44.2)
**Mini-mental state examination, mean (SD)**	8.0 (7.8)
Normal or mild-cognitive impairment (21–30)	18 (7.5)
Moderate-cognitive impairment (10–20)	81 (33.8)
Severe cognitive impairment (< 10)	141 (58.7)
**Delirium**	
Yes	83 (34.6)
No	157 (65.4)
Mini-nutritional assessment, mean (SD)	5.3 (2.3)
**BMI (kg/m**^**2**^**), mean (SD)**	25.2 (4.9)
Underweight (< 18.5)	12 (5.4)
Normal or healthy weight (18.5–24.9)	103 (46.6)
Overweight (25.0–29.9)	69 (31.2)
Obese (≥ 30)	37 (16.7)
**Previous any fractures**	
Yes	94 (39.2)
No	146 (60.8)
**Previous hip fractures**	
Yes	32 (13.3)
No	208 (86.7)
**Type of surgery at baseline**	
Sliding hip screw	23 (9.6)
Intramedullary nail	87 (36.3)
Internal fixation	33 (13.8)
Cemented Hemiarthroplasty	64 (26.7)
Uncemented Hemiarthroplasty	30 (12.5)
Total hip replacement	3 (1.25)

Values are numbers (percentages) unless stated otherwise.

**Figure 1:**
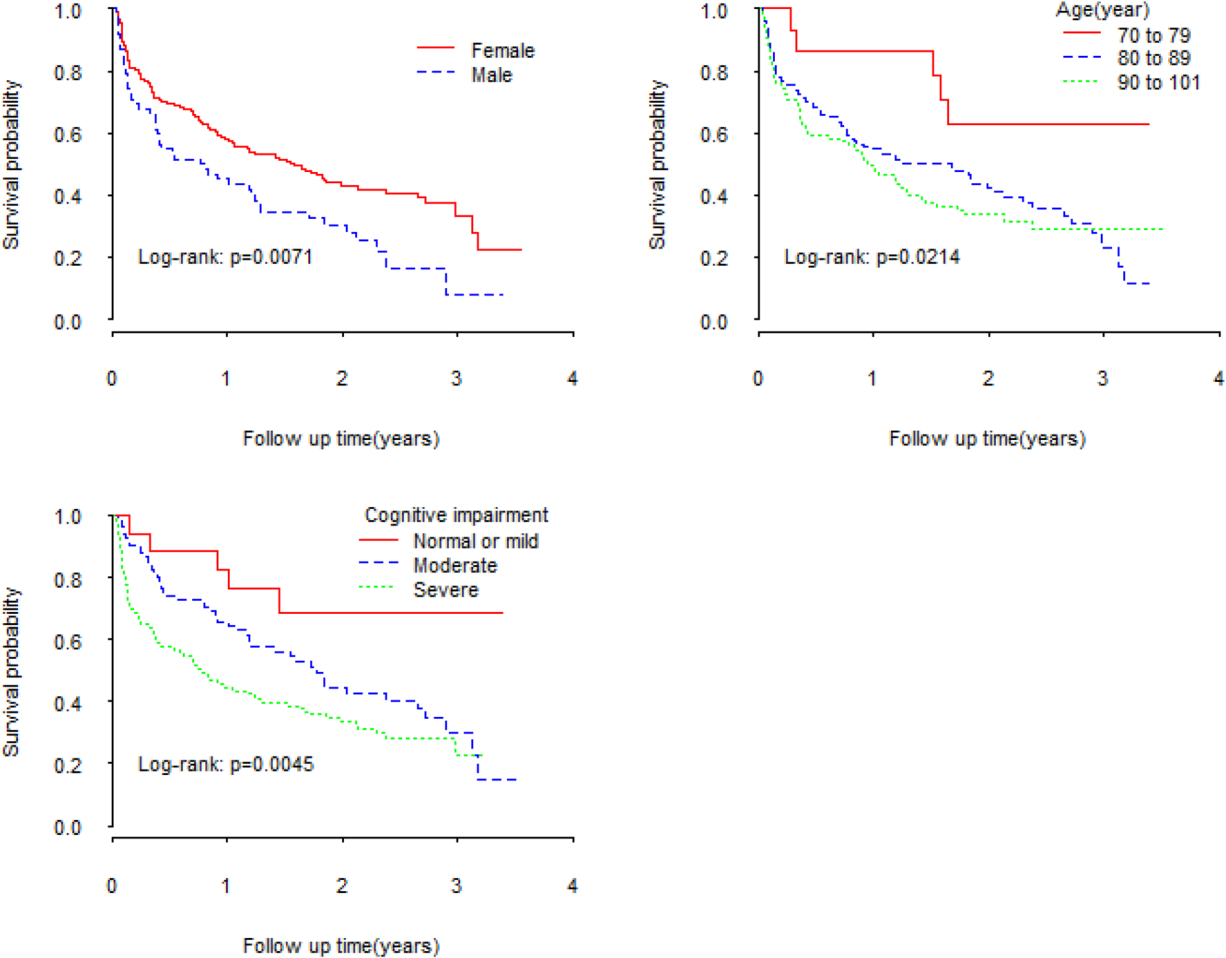
Kaplan–Meier survival plot after hip fracture.

The mean follow-up time for participants was 1.2 years, median was 1.1 years with range from 0.06 to 3.6 years. In total, 149 deaths occurred during the follow-up period. Kaplan–Meier curves (Fig. 1) showed that being female (log rank test: p = 0.0071), being of younger age (log rank: 0.0214) and having a higher MMSE score (log rank test: p = 0.0045) were associated with better survival. The survival curves were steeper (i.e. faster decline) early in the follow up period indicating that mortality rate was highest close to the time of hip fracture. The cumulative death rate within three months was 25.0% (60/240) and the cumulative death rate during the whole study period was 62.1% (149/240). Mortality rate was 118 deaths per 100 person-years within 3 months while the mortality rate of the whole study period (3.6 years) was 51.3 deaths per 100 person-years.

Table 2 showed being female indicated longer life expectancy (coefficient (β) = 0.86, 95% confidence interval (CI):0.39 to 1.34, p = 0.0004). Older age was associated with shorter life expectancy (β = − 0.06, 95% CI: − 0.10 to − 0.02, p = 0.0043). Relative to severe cognitive impairment, normal or mild-cognitive impairment (β = 1.18, 95% CI: 0.05 to 2.31, p = 0.0414) and moderate cognitive impairment (β = 0.56, 95% CI: 0.07 to 1.04, p = 0.0237) were associated with longer life expectancy. Randomisation groups, nutritional status, BMI, previous fractures of any type, previous hip fracture, and different surgery types were not associated with life expectancy.

**Table 2:** Weibull AFT model assessment of the effect of covariates on survival time.

Variables	β (95% CI)	P value
**Group**		
Control	0.35 (−0.10 to 0.80)	0.1276
Intervention	0	
**Gender**		
Female	0.86 (0.39 to 1.34)	0.0004
Male	0	
Age	−0.06 (−0.10 to −0.02)	0.0043
**Mini-mental state examination**		
Normal or mild-cognitive impairment (21 to 30)	1.18 (0.05to 2.31)	0.0414
Moderate-cognitive impairment (10 to 20)	0.56 (0.07 to 1.04)	0.0237
Severe cognitive impairment (< 10)	0	
Mini-nutritional assessment	0.01 (−0.09 to 0.11)	0.8011
**BMI (kg/m**^**2**^**)**		
Obese (≥ 30)	0.60 (−0.09 to 1.29)	0.0864
Overweight (25.0 to 29.9)	0.11 (−0.41 to 0.62)	0.6799
Under weight (< 18.5)	0.11 (−0.81 to 1.03)	0.8103
Normal (18.5 to 24.9)	0	
**Previous any fractures**		
Yes	0.19 (−0.32 to 0.69)	0.4709
No	0	
**Previous hip fractures**		
Yes	0.10 (−0.60 to 0.79)	0.7854
No	0	
**Surgery type**		
Cemented Hemiarthroplasty	−0.004 (−0.73 to 0.73)	0.9992
Internal fixation	0.33 (−0.52 to 1.18)	0.4447
Intramedullary nail	0.03 (−0.68 to 0.74)	0.9352
Sliding hip screw	0.58 (−0.34 to 1.50)	0.2142
Total hip replacement	Not estimable	–
Uncemented Hemiarthroplasty	0	

*AFT* accelerated failure time, *β* coefficient of the Weibull AFT model, *CI* confidence interval, *BMI* body mass index ($\mathrm{weight}$/${\mathrm{height}}^{2}$).

**Figure 2:**
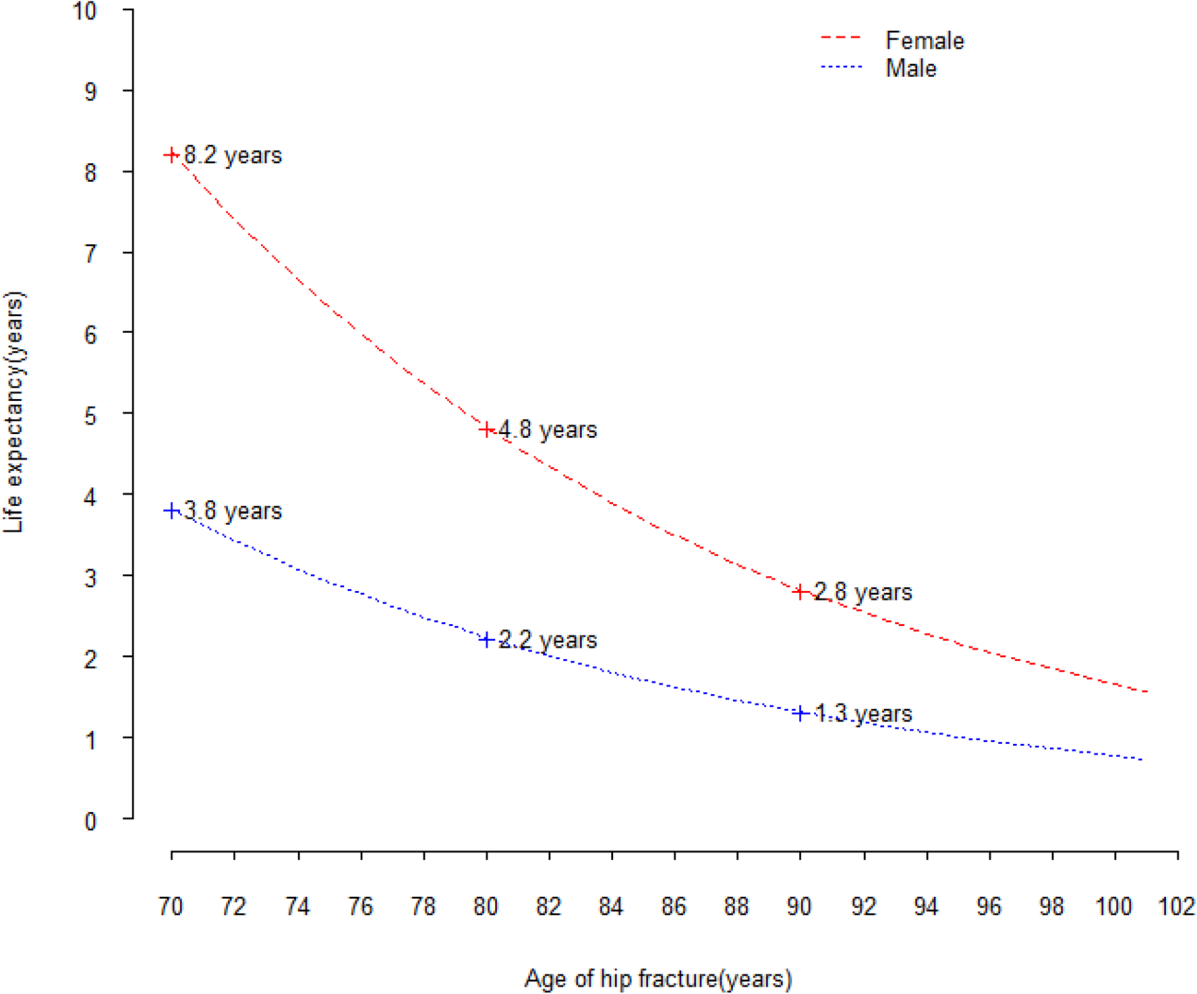
Life expectancy after hip fracture by age and gender.

Using the Weibull AFT model, we calculated life expectancy for different ages by sex. For females who underwent surgical treatment for hip fracture, estimated life expectancy would be 8.2 years for those aged 70 years, 4.8 years for those aged 80 years, and 2.8 years for those aged 90 years. For male patients, estimated life expectancy was 3.8 years for those aged 70 years, 2.2 years for those aged 80 years and 1.3 years for those aged 90 years. (Fig. 2).

Results of sensitivity analysis testing the same covariates in the Weibull AFT model in a multivariable Cox regression model did not yield different results. All estimates were consistent with the Weibull AFT model (in terms of association, direction and significance) (Table 3).

**Table 3:** Sensitivity analysis, Cox proportion model results.

	n	%
**Survival status**		
Alive	91	37.9
Deceased	149	62.1

Predictors	HR (95% CI)	P value
**Group**		
Control	0.76 (0.53 to 1.10)	0.1505
Intervention	1	
**Gender**		
Female	0.51 (0.34 to 0.75)	0.0006
Male	1	
Age	1.05 (1.02 to 1.08)	0.0043
**Mini-mental state examination**		
Normal or mild-cognitive impairment (21 to 30)	0.37 (0.15 to 0.94)	0.0356
Moderate-cognitive impairment (10 to 20)	0.64 (0.43 to 0.95)	0.0256
Severe cognitive impairment (< 10)	1	
Mini-nutritional assessment	0.99 (0.91 to 1.07)	0.7433
**BMI (kg/m**^**2**^**)**		
Obese (≥ 30)	0.85 (0.39 to 1.82)	0.6680
Overweight (25.0 to 29.9)	0.94 (0.61 to 1.43)	0.7539
Under weight (< 18.5)	0.63 (0.36 to 1.10)	0.1046
Normal (18.5 to 24.9)	1	
**Previous any fractures**		
Yes	0.86 (0.56 to 1.30)	0.4695
No	1	
**Previous any fractures**		
Yes	0.92 (0.52 to 1.63)	0.7787
No	1	
Surgery type		
Cemented hemiarthroplasty	0.98 (0.54 to 1.80)	0.9582
Internal fixation	0.79 (0.39 to 1.59)	0.5100
Intramedullary nail	0.97 (0.54 to 1.74)	0.9161
Sliding hip screw	0.62 (0.29 to 1.34)	0.2243
Total hip replacement	–	0.9784
Uncemented hemiarthroplasty	1	

*HR* hazard ratio, *CI* confidence interval, *BMI* body mass index ($\mathrm{weight}$/${\mathrm{height}}^{2}$).

## Discussion

In this longitudinal study of 240 hip fracture patients, we found that older age, male gender and greater impairment of cognition were associated with reduced life. Life expectancy of hip fracture patients living in aged care facilities was lower than in the general Australian population especially for those younger patients. This study also found that mortality rate was much higher immediately following the hip fracture.

Life expectancy is defined as the average number of years a group of people is expected to live at a certain age, comparing hip fracture patients’ life expectancy with that of the general population could show a straightforward impact of hip fracture on the population and the disease burden. The Treasurer of the Commonwealth of Australia estimated that in 2015 for a 70 year old female the life expectancy was 19.3 years (i.e. at 70 years old she was expected to live to 89.3 years) and for a 70 year old male the life expectancy was 16.9 years^19^. Therefore, the life expectancy of a 70 year old female hip fracture patient living in an aged care facility could be 11 (19.3–8.2 = 11.1) years shorter than that of the Australian general population, and it could be 13 years (16.9–3.8 = 13.1) shorter for a 70 year old man.

Studies have shown that women had a much higher risk for hip fracture than men, but men had much higher mortality than women after hip fractures^20–24^. In our study we observed that men’s hazard rate for death was about twice that of women (HR = 1.98). Our study also found that if a hip fracture happened at a younger age, males would have much shorter life expectancy than females, however the difference decreased at older ages. Previous studies have found cognitive impairment to be a major risk factor for mortality after hip fracture and this was confirmed by our study^25,26^. Studies showed MMSE cannot be used to detect delirium but can be used to ruling out delirium as shown by several studies^27,28^. Our data also support that MMSE can be used to rule out delirium, such as with normal cognitive (MMSE > 24) none of the patients had delirium, however among severe cognitive impairment (MMSE < 10) patients about 50% patients had delirium which means high MMSE will be useful to rule out delirium. However, to avoid collinearity, we did not put delirium variable into the final model since the delirium was highly correlated with MMSE (Spearman correlation coefficient = − 0.87, p < 0.0001). The relationship between mortality and BMI after hip fracture was not consistent; some studies suggested that being overweight or obese were protective factors for mortality after hip fracture^29,30^, however, Akinleye et al. followed 15,108 patients who underwent surgery for hip fracture over a 5 year period and found that either extreme of the BMI spectrum had the highest mortality rates^31^. In our study we did not find an association between BMI and mortality after hip fracture in these older patients. We did not find any association between the type of hip surgery and life expectancy, this result was consistent with a retrospective registry-based cohort study of 14,932 patients undergoing hip fracture surgery in Sweden^32^.

Mortality rate after hip fracture is associated with time since injury. Kanis et al. showed that mortality rate after a hip or vertebral fracture is non-linear; the rate slows down significantly with time^33–35^. We found the same phenomenon as the mortality rate within three months was much higher than the whole study period. However, a study showed that death rate was increasing in the first 6 to 9 months after hip fracture due to infection and cardiovascular disease^36^. Interventions at which stage and what kind of interventions can more effectively prevent excessive deaths warrant further studies.

This study had several strengths. The commencements of follow up and baseline characteristics of the patients were well defined and accurately measured. The follow-up rate was high and patients who were lost follow-up and withdrew from the study were well documented. Our study directly calculates the survival time which is the most natural measure of life for both clinicians and patients. This study had limitations. Firstly, for the clinical trial, 2210 patients were aassessed for eligibility, 1766 were not meeting inclusion criteria and 114 declined to participate which made our sample size as 240, this inclusion/exclusion procedures made our study lack of generalisability and our results might not be generalized to broader hip fracture patients. Secondly, the life expectancy in this study was calculated by parametric method while the life expectancy of Australian population was calculated by life table method, the parametric method could overestimate the life expectancy of the patients and cause extra bias when comparing the life expectance between the specific population and the general population^37^. Thirdly, our sample size was small and we did not provide confidence intervals for the life expectancies, further studies with bigger sample size might provide more accurate life expectancies for hip fracture patients. Finally, the follow up time of this study was short, our model parameters derived from the short period of follow up time might not fit long term mortality of the population.

## Conclusions

Overall, our data suggested that age, gender and cognition were associated with life expectancy of hip fracture patients. The life expectancy of hip fracture patients living in aged care facilities was much lower than that of the general Australian population as would be expected with this very disabled group of older people. Hip fracture occurring at a younger age in this population could cause more loss of life expectancy. Interventions at which stage and what kind of interventions can more effectively prevent excessive deaths warrant further studies.

## Data Availability

The data that support the findings of this study are available from Dr. Enwu Liu or Professor Maria Crotty (Maria.Crotty@sa.gov.au), upon reasonable request and ethnic approval from the Southern Adelaide Clinical Human Research Ethics Committee.
